# Selective Regional Alteration of the Gut Microbiota by Diet and Antibiotics

**DOI:** 10.3389/fphys.2020.00797

**Published:** 2020-07-07

**Authors:** Elesa Poteres, Nathaniel Hubert, Sudeep Poludasu, Gabriella Brigando, Julia Moore, Kelly Keeler, Allison Isabelli, Iara Cassandra V. Ibay, Lauren Alt, Matthew Pytynia, Mae Ciancio, Kristina Martinez-Guryn

**Affiliations:** Laboratory of Dr. Martinez-Guryn, Midwestern University, College of Graduate Studies, Biomedical Sciences Department, Downers Grove, IL, United States

**Keywords:** gut microbiota, antibiotics, gastrointestinal regions, diet, microbial diversity, small intestine

## Abstract

The small intestinal microbiota has recently been implicated in contributing to metabolic disease. We previously demonstrated that diets rich in saturated milk fat have a particularly strong impact on the small bowel microbiota as opposed to more distal gastrointestinal (GI) regions. However, the impact of antibiotics and diet on the small bowel microbiota has not been clearly demonstrated. Thus, we sought to determine how diet and antibiotics interact in modulating the regional landscape of the gut microbiota. We conducted a study using male mice on a high fat (HF) or a low fat (LF) diet (*n* = 15/group) that received either water control (*n* = 5/diet), rifaximin, (non-absorbable broad-spectrum antibiotic; *n* = 5/diet) or an antibiotic cocktail consisting of metronidazole, cefoperazone, vancomycin, and neomycin (Abx cocktail; *n* = 5/diet). 16S rRNA sequencing was performed on mucosal scrapings collected from the small intestine and cecum, as well as on stool samples. Interestingly, antibiotics had a significant effect on community composition throughout the small intestine, cecum and stool, whereas diet significantly affected only the jejunum and cecum microbiota. The antibiotic cocktail, regardless of diet, was most effective in increasing cecum size, reducing body fat percentage, and plasma lipid levels. Altogether, this study reveals a selective and divergent regional alteration of the gut microbiota by diet and antibiotics.

## Introduction

Various factors influence microbial composition and alter colonization including diet, genetics, age, and antibiotics (Fujisaka et al., 2016). Dietary effects on gut microbiota composition have been a major focus in gut microbiota research but most studies have primarily concentrated on the colonic portion of the gut. However, more recent research has shown that diet alters small intestinal microbiota (Martinez-Guryn et al., 2018). For example, we previously showed that a diet rich in saturated milk fat selectively altered the jejunal and ileal microbiota composition. Jejunal microbiota transplant from HF-fed mice increased lipid absorption in germ free (GF) mice compared to microbiota from LF-fed mice. Thus, modulation of small intestinal bacterial composition influences nutrient absorption and metabolic profiles (Mu et al., 2017; Martinez-Guryn et al., 2018).

Small intestinal bacteria aid in the absorption of nutrient-derived carbohydrates, vitamins, lipids, and amino acids, as well as synthesis of certain micronutrients (Sundin et al., 2017; Adike and DiBaise, 2018), while colonic microbes aid in the fermentation of undigested carbohydrates into short-chain fatty acids (Adike and DiBaise, 2018), important in epithelial cell health (Kelly et al., 2015). Altered pH, oxygen levels, foodstuffs, increased motility and bile acids are a few examples that distinguish the small bowel environment from the distal gut. This results in a widely different microbial composition by region through the small intestine, cecum and stool (reviewed in Tropini et al., 2017; Martinez-Guryn et al., 2019). Given that microbial community structures and functions differ along the length of the gut, it is reasonable to consider that differential effects would be observed based on specific environmental factors including dietary intake and antibiotic treatments commonly used to treat intestinal disorders.

How antibiotics impact host-microbial balance along the gastrointestinal tract under conditions of low and high fat dietary intake has not been clearly established. Neither an evaluation of the interactive effects of a high fat diet in conjunction with antibiotic treatment on the small bowel microbiota, nor a comparative analysis of the effectiveness of antibiotics versus diet along the length of the gut have been previously reported. However, these considerations may be important for those suffering from the ill effects of intestinal diseases that alter both nutrient absorption and metabolism as well as host-microbial balance.

To address this need, we performed a study examining how diet in combination with two different antibiotic protocols impacted the regional gut microbiota. We characterized the microbial composition present in the mucosa of the duodenum, jejunum, ileum, and cecum, as well as stool.

For this study, mice were fed a high fat (HF; 18%) or low fat (LF; 4%) diet for 4 weeks (*n* = 15/group). Each group received either water control (*n* = 5/diet), rifaximin (*n* = 5/diet), or an antibiotic cocktail consisting of metronidazole, cefoperazone, vancomycin, and neomycin (*n* = 5/diet). Our goal was to examine how gut microbial composition could be altered not only from these select antibiotics, but also from the interaction of antibiotics and diet, in different regions of the gut. We hypothesized that diet would specifically impact the small bowel microbiota and antibiotics would alter the gut microbial composition along the various regions of the gut. We demonstrated that diet had a significant and specific impact on the jejunal and cecal microbiota compositions, whereas antibiotics impacted all regions of the gut examined. An interaction between diet and antibiotics reached significance only in the jejunum and ileum in altering microbial composition. The regional selectivity of HF diets and potential interactions with antibiotics may offer unique insights into mechanisms impacting host-microbial balance and thus more effective treatment options for those suffering from the debilitating effects of intestinal dysbiosis.

## Materials and Methods

### Animals

This study was approved by the Midwestern University Institutional Animal Care and Use Committee. C57BL/6 male mice were purchased from Jackson Laboratory (Mount Desert Island, Maine). Upon receipt, 30 mice (7,8 weeks old) were acclimated to the facility for one week. Next, bedding was mixed across all mice prior to the start of the study to normalize the microbiota and mice were also individually housed, one mouse per cage. Mice were randomized into either an experimental high fat (HF; *n* = 15; 18% anhydrous milkfat by weight; Harlan Teklad TD.97222) or low fat (LF; *n* = 15; 4% fat; Harlan Teklad TD.00102) diet control group. Diet compositions have been previously published (Martinez-Guryn et al., 2018). One week after diet introduction, mice in both groups were treated for 4 weeks via oral gavage, 5 times per week, with 200 μl of either a water vehicle control, rifaximin (30 mg/kg), or an antibiotic cocktail [(30 mg/kg) consisting of metronidazole (10 mg/kg), cefoperazone (10 mg/kg), vancomycin (5 mg/kg) and neomycin (5 mg/kg)]. Each group consisted of five mice per group. Body weight, food intake, and stool were collected weekly for the duration of the study. Mice were euthanized by CO_2_ followed by cervical dislocation. Mesenteric, epididymal, retroperitoneal and inguinal fat pads were weighed, cecum lengths were measured, and the tissues and plasma were snap frozen and stored at −80°C for downstream analyses. Beginning at the base of the stomach, the proximal 4 cm of the small intestine was collected as the duodenum. The next 2 cm were disposed, and the following 6 cm were collected as the jejunum. Ileum was harvested beginning at 8 cm proximal to the cecum where the most distal 2 cm were disposed (6 cm total). Cecum length was measured in centimeters and in some cases only an *n* = 3 was obtained per group due to missing measurements during the animal harvest. Segments of the small intestine and cecum were lightly scraped with glass slides to remove luminal contents and mucosa was collected for further analyses.

### Plasma Lipids

Plasma triglyceride and low density lipoprotein levels were measured using commercial colorimetric test kits from Wako Chemicals (Richmond, VA, United States) following manufacturer’s microplate protocols.

### DNA Extraction and 16S rRNA Amplicon Sequencing

DNA was extracted using previously published protocols (Wang et al., 2009). DNA collected from the small intestinal and cecal mucosal scrapings and stool was sent to Argonne National Laboratory for 16S rRNA amplicon sequencing conducted via Illumina Mi-Seq. Quality filtering and downstream analyses were performed using previous protocols (Martinez-Guryn et al., 2018). Any sequences that were found as unknown were blasted to determine the origin and if determined to be eukaryotic were removed from the analysis. Briefly, Illumina-utils software was used to dumultiplex, merge paired reads, and quality filter sequence data. Minimum entropy decomposition was used to assess quality filtered reads and high-resolution oligotypes were generated using Shannon entropy (Eren et al., 2013). Taxonomy was assigned to oligotypes using GAST as previously described (Martinez-Guryn et al., 2018). Shannon diversity and observed species analyses were performed using QIIME software and shown for each intestinal region, diet, and antibiotic group (Figures 3A,B). The max number of sequences obtained in a sample was 38,327 and the average sequencing depth was 13,568.14. In general, fewer sequences were obtained from the upper GI regions (duodenum 4596.54; jejunum 7157.9; ileum 7934.19; cecum 19523.1; stool 27452.3). Rarefaction was not performed prior to alpha and beta diversity comparisons based on several lines of evidence showing that rarefaction leads to loss of valuable information and precision, thereby reducing sensitivity and reproducibility (Fernandes et al., 2014; McMurdie and Holmes, 2014). Based on Bray Curtis dissimilarity index, a PCoA plot was generated on a forced axis for intestinal region utilizing QIIME (Figure 3 and Supplementary Figures 1C,D, and Supplementary Figure 2) or on a forced axis for diet or antibiotics (Supplementary Figures 1A,B). A two-way Adonis test was performed in QIIME to assess significance between antibiotics and diet based on Bray Curtis metrics as well as interactive effects between diet, antibiotics and intestinal region (Supplementary Table 1) (Caporaso et al., 2010). Post-hoc pairwise comparisons were made with pairwise Adonis software in R version 0.4 shown in Supplementary Table 2 (Martinez Arbizu, P. 2020). Anvi’o was used to generate heat maps displaying taxonomic differences within diet and antibiotic groups (Figure 4). The normalized relative taxonomic abundance at the family level was compared via Kruskal-Wallis in QIIME based on diet and antibiotic treatment within each gut region (FDR correction *p* ≤ 0.05) on QIIME software (see Supplementary Table 3). In some cases, groups contained an *n* = 3 due to samples failing the sequencing run or having too few sequences to be incorporated into the analysis. Samples with less than 250 sequences were removed from the analysis.

### 16S rRNA Gene Quantification

16S rRNA gene copy number was determined from small intestinal and cecum mucosal scrapings or stool as previously described (Martinez-Guryn et al., 2018). Genes were quantified by determining a standard curve for gene copy number by cloning primer sequences into pCR4-TOPO plasmids. Forward (F) and reverse (R) primer sequences for 16S rRNA: F-TCCTACGGGAGGCAGCAGT; R-GGACTACCAGGGTATCTAATCCTGTT.

### Statistical Analyses

**FIGURE 1:**
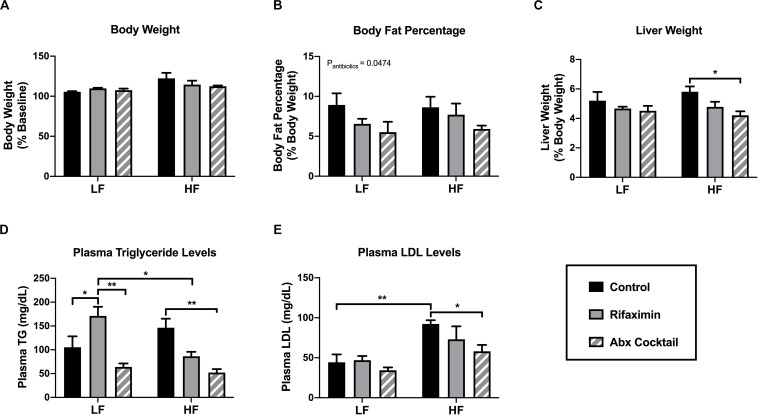
Antibiotic-treated mice display reduced body fat and plasma lipid levels. C57Bl6 mice were fed a low (LF) or high fat (HF) diet and treated with vehicle control, rifaximin or an antibiotic (Abx) cocktail for 4 weeks. **(A)** Body weight was measured and expressed as percentage from baseline. **(B)** Body fat mass was estimated based on the summation of fat pad weights expressed as a percentage of body weight. Liver weight was measured in grams and then expressed as a percentage of total body weight **(C)**. Plasma triglyceride **(D)** and low density lipoprotein were measured **(E)**. Data are shown as means ± SEM; *n* = 5 per group; *p < 0.05, **p < 0.01.

**FIGURE 2:**
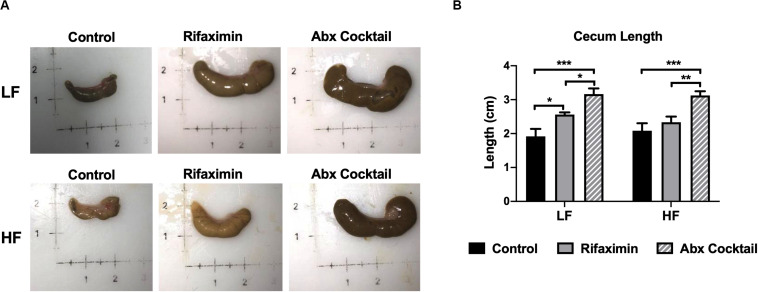
Antibiotic-treated mice display enlarged cecums. C57Bl6 mice were fed a low (LF) or high fat (HF) diet and treated with vehicle control, rifaximin or an antibiotic (Abx) cocktail for 4 weeks. **(A)** Pictures of representative ceca are shown. **(B)** Cecum length was measured and reported in centimeters. Data are shown as means ± SEM; *n* = 3,4 per group; *p < 0.05, **p < 0.01, ***p < 0.001.

**FIGURE 3:**
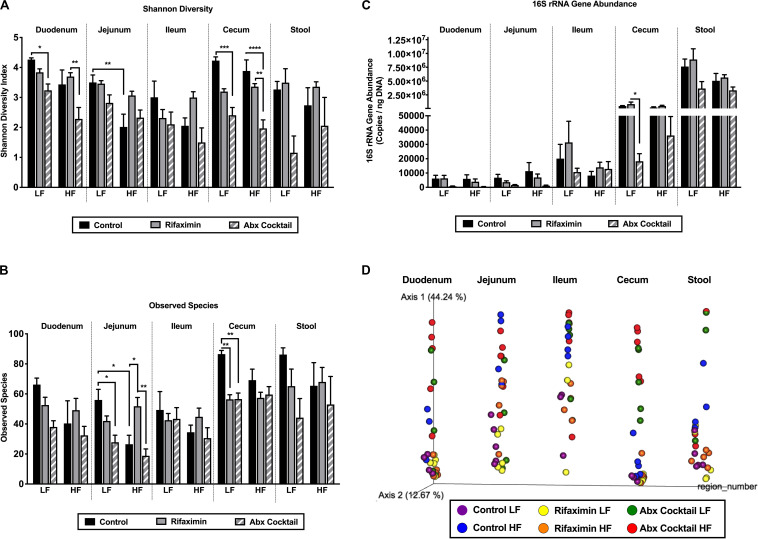
Diet and antibiotics differentially impact the gut microbiota along the length of the gut. C57Bl6 mice were fed a low fat (LF) or high fat (HF) diet and treated with vehicle control, rifaximin or an antibiotic (Abx) cocktail for 4 weeks. **(A)** Shannon diversity is shown for all gut regions. **(B)** The number of observed species is shown. **(C)** 16S rRNA gene abundance is shown. **(D)** A PCoA plot based on Bray Curtis dissimilarity index and on a forced axis for region is shown. See Supplementary Figure 1 for PCoAs ploted on a forced axis for diet and antibiotics and Supplementary Figure 2 for diet comparisons within each antibiotic treatment condition. Results from two-way adonis tests are shown in Supplementary Table 1 and pairwise comparisons are shown in Supplementary Table 2. Data are shown as means ± SEM; *n* = 5 per group; *p < 0.05, **p < 0.01, ***p < 0.001, ****p < 0.0001.

Statistical analyses were performed using GraphPad Prism v8.0; a two-way ANOVA was employed followed by a Tukey’s multiple comparisons post-hoc test examining all possible comparisons as well as the simple effects of antibiotics within each diet for Figures 1–3. Data are presented as means +/− SEM and differences between group means were considered significant at *p* ≤ 0.05. Unless otherwise indicated, there was an *n* = 5 per group and outliers were removed based on the ROUT outlier detection method.

## Results

### The Antibiotic Cocktail Reduced Body Fat Percentage and Plasma Lipid Levels

To determine the impact of rifaximin or the antibiotic cocktail on general metabolic outcomes, body weight, liver weight, adiposity, and plasma lipid levels were measured. Body weight was not influenced by antibiotic treatment under either dietary condition (Figure 1A), nor was food intake. Averaged food intake was 12.71 kcal/mouse/day and 13.09 kcal/mouse/day for the LF and HF mice, regardless of antibiotic treatment (data not shown). To determine the effect of the antibiotic cocktail on body fat percentage in the mice, four fat pads were taken from each mouse, including mesenteric, gonadal, inguinal and retroperitoneal fat pads, and their masses summed to represent total fat percentage compared to total body weight. Mice treated with the antibiotic cocktail displayed a significant reduction in body fat percentage (main effect of antibiotic cocktail compared to control, *p* < 0.05; Figure 1B) and liver weight in mice fed a HF diet compared to control (Figure 1C). Consistently, treatment with the antibiotic cocktail reduced plasma triglyceride (Figure 1D) and low density lipoprotein (LDL; Figure 1E) levels compared to controls under HF diet conditions, only. An interactive effect of diet and antibiotic treatment was observed in altering plasma triglyceride levels (interaction: *p* = 0.0043, diet: *p* = ns, antibiotics: *p* = 0.0008). Taken together, the antibiotic cocktail resulted in a lean phenotype in the HF-fed mice compared to rifaximin-treated and control animals.

### Rifaximin and Antibiotic Cocktail Increase Cecum Size

Host features indicative of reduced bacterial load were examined by assessing cecum size and length, which are reported to be grossly enlarged in germ-free animals (Devkota et al., 2012). As shown in Figure 2, cecum size was increased by rifaximin and antibiotic cocktail compared to controls. Cecum length was significantly increased by the antibiotic cocktail compared to controls under both LF and HF conditions (Figure 2B). Rifaximin increased cecum length under LF but not HF feeding groups. The antibiotic cocktail was more effective in causing enlarged ceca which may be due to a reduction in total bacterial load.

### Diet and Antibiotics Have a Divergent Impact on the Regional Gut Microbiota

In order to determine diet and antibiotic-mediated shifts in the gut microbiota, alpha and beta diversity analyses were performed as well as heat maps generated for depicting changes in taxonomy. First, Shannon diversity is shown in Figure 3A which takes into account richness and evenness of microbial communities. There was a main effect of antibiotics on Shannon diversity in the duodenum (*p* = 0.0002), jejunum (*p* = 0.0246), cecum (*p* = 0.0001), and stool (0.0086), but not in the ileum. Specifically, the antibiotic cocktail reduced Shannon diversity in the duodenum and cecum under LF conditions compared to the control group. The antibiotic cocktail also reduced Shannon diversity in the cecum under HF conditions in the cecum (Figure 3A). Regarding the dietary impact, a main effect of diet was observed on Shannon Diversity in the duodenum (*p* = 0.0037) and jejunum (*p* = 0.0007), but not in the ileum, cecum, or stool. In addition, a trend for an interaction between diet and antibiotics was only observed in the jejunum (*p* = 0.0764), but no other regions of the gut. In the jejunum, HF diet reduced Shannon diversity compared to the LF diet control. Taken together, Shannon diversity is impacted by region, antibiotics, and diet, with antibiotics being most effective in reducing Shannon diversity in the duodenum and cecum and less effective in the jejunum and ileum, but with a trend for an interaction between diet and antibiotics in the jejunum.

The number of observed species, which factors in richness but not evenness, is shown in Figure 3B. In general, the number of observed species was lower in the upper small intestine compared to the cecum and stool. The antibiotic cocktail reduced the number of observed species compared to control under LF diet conditions in the jejunum and the cecum. HF diet reduced the number of observed species compared to the LF diet control in the jejunum. Lastly, 16S rRNA gene abundance was measured in each gut region (Figure 3C). As expected, 16S rRNA gene content increased along the length of the gut with the greatest amount found in the stool (Figure 3C). 16S rRNA gene abundance was significantly reduced by the antibiotic cocktail under LF diet in the cecum compared to control (Figure 3C).

Changes in beta diversity were examined using the Bray Curtis dissimilarity index depicted in the PCoA plots in Figure 3D and Supplementary Figures 1, 2. The PCoA plot in Figure 3D was generated on a forced axis for gut region to provide a comparison of all regions of the gut examined. Within each region, diet and antibiotic group comparisons are shown. Herein, it appeared that HF diet alters the jejunal microbiota composition, whereas community composition largely differs in the cecum by antibiotic treatment. Adonis statistical tests (Supplementary Table 1) revealed a significant impact of diet on gut microbiota composition in the jejunum (*R*^2^ 0.2228, *p* = 0.001) and cecum (*R*^2^ 0.0445, *p* = 0.03). Antibiotics significantly impacted all regions of the gut including the duodenum (*R*^2^ 0.0737, *p* = 0.001), jejunum (*R*^2^ 0.0823, *p* = 0.002), ileum (R^2^ 0.1467, *p* = 0.048), cecum (*R*^2^ 0.0343, *p* = 0.001), and stool (*R*^2^ 0.0762, *p* = 0.002). Trends toward an interaction between diet and antibiotics were observed in the jejunum (*R*^2^ 0.0823, *p* = 0.092) and ileum (*R*^2^ 0.1467, *p* = 0.061). Supplementary Figures 1, 2 offer different views of diet-antibiotic interactions. Supplementary Figures 1A,B depict all samples but on a forced axis for diet and antibiotic treatment, respectively. The PCoA in Supplementary Figure 1A shows that the impact of the antibiotic cocktail is more influential in LF compared to HF diet conditions. The PCoA in Supplementary Figure 1B demonstrates that the HF diet effect on community composition is most clear in the control mice compared to mice treated with rifaximin or the antibiotic cocktail. In Supplementary Figure 2, PCoAs were generated to separate out samples from control, rifaximin, or antibiotic-treated mice to show the dietary effects within each treatment condition across the various regions of the gut. Based on these orientations, it is also evident that HF diet impacts community composition in control conditions but less so with treatment of rifaximin and the antibiotic cocktail.

**FIGURE 4:**
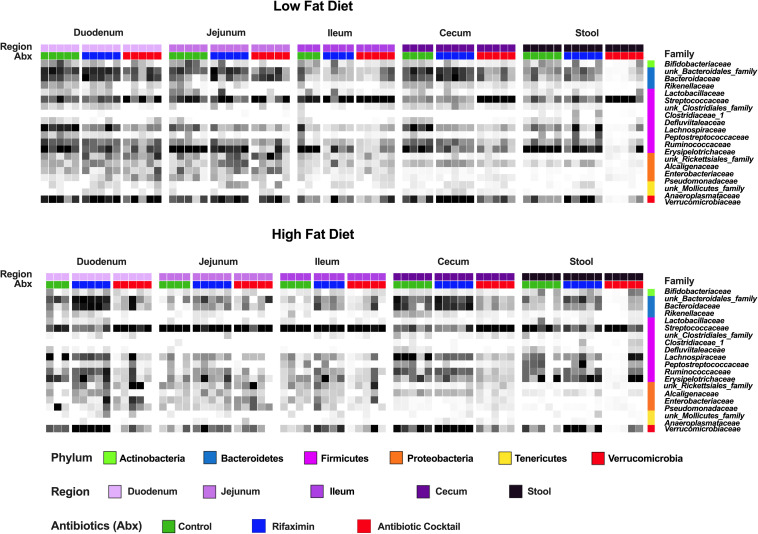
Diet and antibiotics differentially impact the relative abundance of gut microbial taxa along the length of the gut. C57Bl6 mice were fed a low fat (LF) and high fat (HF) diet and treated with vehicle control, rifaximin or an antibiotic cocktail for 4 weeks. The relative abundance of different taxa are illustrated. Note the relative change in abundance as a function of diet in the jejunum. Note the relative change in abundance as a function of antibiotic treatment in the stool and cecum. Data shown are *n* = 3–5 per group.

Heat maps displayed in Figure 4 reveal relative abundances of bacteria across all treatment conditions at family level. It is evident that antibiotic cocktail treatment decreased the abundance of several families in the cecum as compared to other regions of the gut. Whereas, the dietary impact is more evident in the jejunum in which the HF diet reduced the abundance of several families compared to LF diet, with the exception of Streptococcaceae that was increased in the by HF vs LF diet. Less notable changes of diet were apparent in other regions of the gut. Kruskal Wallis statistical tests were performed for identifying taxa altered between diet and treatment groups and shown in Supplementary Table 3. Altogether, diet appeared to have a specific impact on the jejunal microbiota, whereas antibiotics impacted all regions of the gut, with the most pronounced effect in the duodenum and cecum based on observed changes in taxa abundance (Figure 4) as well as from beta and alpha diversity analyses (Figure 3).

## Discussion

We demonstrated that diet and antibiotics have divergent, yet potentially interactive effects in different regions of the gastrointestinal (GI) tract. The jejunum is a major site for diet-induced changes in gut microbiota structure, whereas the cecum is a key site for antibiotic-mediated changes in gut microbiota composition. Overall it appeared that antibiotic treatment (rifaximin or antibiotic cocktail) obscured the dietary impact on the gut microbiota in various regions of the gut (Supplementary Figure 2). Studies in the literature have independently examined antibiotic or diet effects along the length of the gut (Mu et al., 2017; Martinez-Guryn et al., 2018), but to our knowledge, this is the first report examining both factors and their interaction in different regions of the gut.

Intestinal diseases, such as irritable bowel syndrome, inflammatory bowel disease, and small intestinal bacterial overgrowth (SIBO) are routinely treated with antibiotics. Drugs such as rifaximin, metronidazole, and neomycin, among others, are used to ease symptoms in an attempt to restore microbial eubiosis (Shayto et al., 2016; Adike and DiBaise, 2018; Nogueira et al., 2019; Saffouri et al., 2019). In SIBO, malabsorption occurs secondary to changes in host-microbial balance (Adike and DiBaise, 2018). Antibiotics are typically the main treatment modality for this condition (Fujisaka et al., 2016; Adike and DiBaise, 2018), with rifaximin being the most widely recommended (Adike and DiBaise, 2018). Previous studies examining short or long term use of rifaximin demonstrated significant alterations in the gut microbiota, with preferential changes in the small intestinal luminal contents (Kim et al., 2013; Mu et al., 2017). While rifaximin is known to have localized versus peripheral effects (Kim et al., 2013), in our study rifaximin increased plasma triglyceride levels in LF-fed mice to levels observed in HF-fed control mice. Thus, understanding the interaction between intestinal microbes, diet, and antibiotics would be useful in the clinical setting for selecting more targeted therapeutics.

Recent research has shown site-specific effects of antibiotics. In a study conducted in pigs, antibiotics (50 mg/kg olaquindox, 50 mg/kg oxytetracycline calcium, 50 mg/kg kitasamycin; antibiotic treatment group from 23 to 42 days of age) altered gut microbiota composition in more distal vs proximal regions of the GI tract (Mu et al., 2017). Similar to our findings, Mu et al. (2017) found that the ileum has reduced microbial diversity compared to other regions of the gut, which is perhaps due to greater host immune pressure in this region (Martinez-Guryn et al., 2019). Differences in diversity along the length of the gut appear to span both murine and porcine animal models and may apply to other host species. Another report in mice demonstrated an interaction of a fiber-deficient diet in the recovery from antibiotic treatment, albeit these analyses were based on stool (Ng et al., 2019). Further investigation into varying types of diet either high in fat or fiber-free is warranted for a better understanding of potential interactions in altering the gut microbiota along with antibiotic use as well as the mechanisms involved. Bile acids represent a possible mechanism through which diet may elicit regional changes in gut microbiota structure and may be the crux of diet-antibiotic interactions. While bile acid levels and composition were not measured in this study, the role of bile acids in this process will be a goal of future research.

Even though a limitation to our study is the small sample size, significant effects and interactions were found with both dietary and antibiotic treatments on the outcomes measured. We offer a direct comparison of antibiotics and diet showing that these factors have a selective regional impact on the gut microbiota. The results from this research may represent a step towards personalized medicine: specific antibiotics may have differing effectiveness depending on their intended target along the intestinal tract and according to differences in individual diet history. Dietary interactions with antibiotics should be further evaluated in the treatment of intestinal diseases where antibiotic use is a common therapeutic intervention.

## Data Availability Statement

The sequencing data generated in this study has been deposited into the bioproject database (accession: PRJNA594399).

## Ethics Statement

The animal study was reviewed and approved by Midwestern University Institutional Animal Care and Use Committee.

## Author Contributions

EP, KM-G, MP, and MC conceptualized the study design. EP, KM-G, GB, SP, NH, and KK wrote the manuscript. EP, JM, MP, AI, KK, II, LA, and KM-G performed the experiments. EP, KM-G, and NH analyzed the data and performed statistical analyses. All authors contributed to editing the manuscript and approved the submitted version.

## Conflict of Interest

The authors declare that the research was conducted in the absence of any commercial or financial relationships that could be construed as a potential conflict of interest. The reviewer VL delcared a past co-authorship with one of the authors KM-G to the handling Editor.
