# Supplementary material for: Selective Regional Alteration of the Gut Microbiota by Diet and Antibiotics
Source: Front Physiol. 2020 Jul 7;11:797. doi: 10.3389/fphys.2020.00797 (PMC7358400; doi:10.3389/fphys.2020.00797)
Supplement: Supplementary file 1 [file Data_Sheet_1.docx]

Supplementary Material

# Supplementary Figures

**
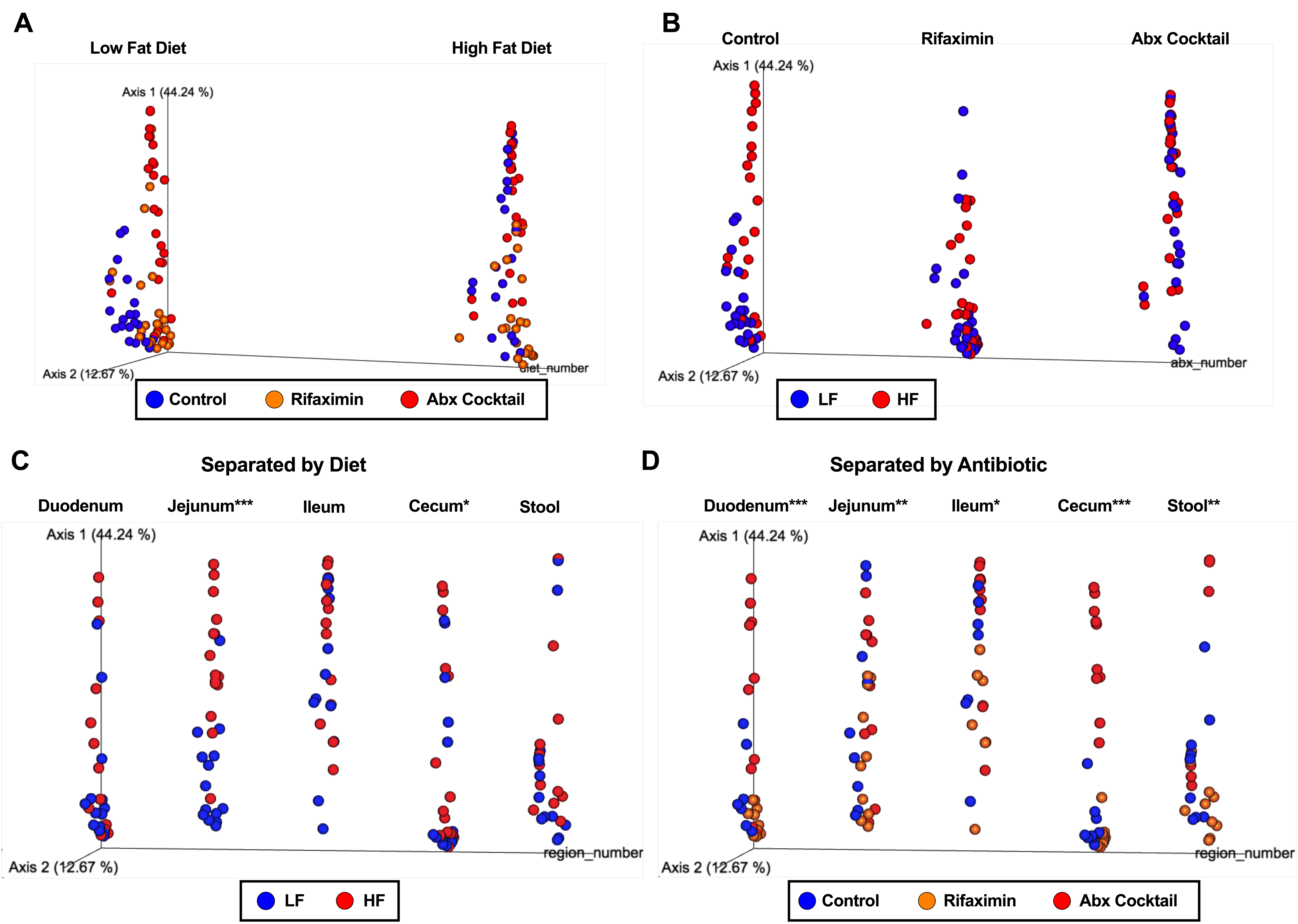
**

**Supplementary Figure 1.** **Diet and antibiotics differentially impact the gut microbiota along the length of the gut.** C57Bl6 mice were fed a low fat (LF) or high fat (HF) diet and treated with vehicle control, rifaximin or an antibiotic (Abx) cocktail for 4 weeks. A) A PCoA plot based on Bray Curtis dissimilarity index and on a forced axis for diet is shown to illustrate the impact of antibiotic treatment under either dietary condition. B) A PCoA on a forced axis for antibiotics is shown to illustrate the impact of diet within each antibiotic treatment condition. C-D) PCoA plots based on Bray Curtis dissimilarity index and on a forced axis for region is shown and categorized by diet (C) or antibiotic (D) treatment. Adonis tests were performed to establish significant differences based on the main effects of diet (C) or antibiotics (D), * p < 0.05, ** p < 0.01, *** p < 0.001 (see also Supplemental Table 1 for adonis results summary).


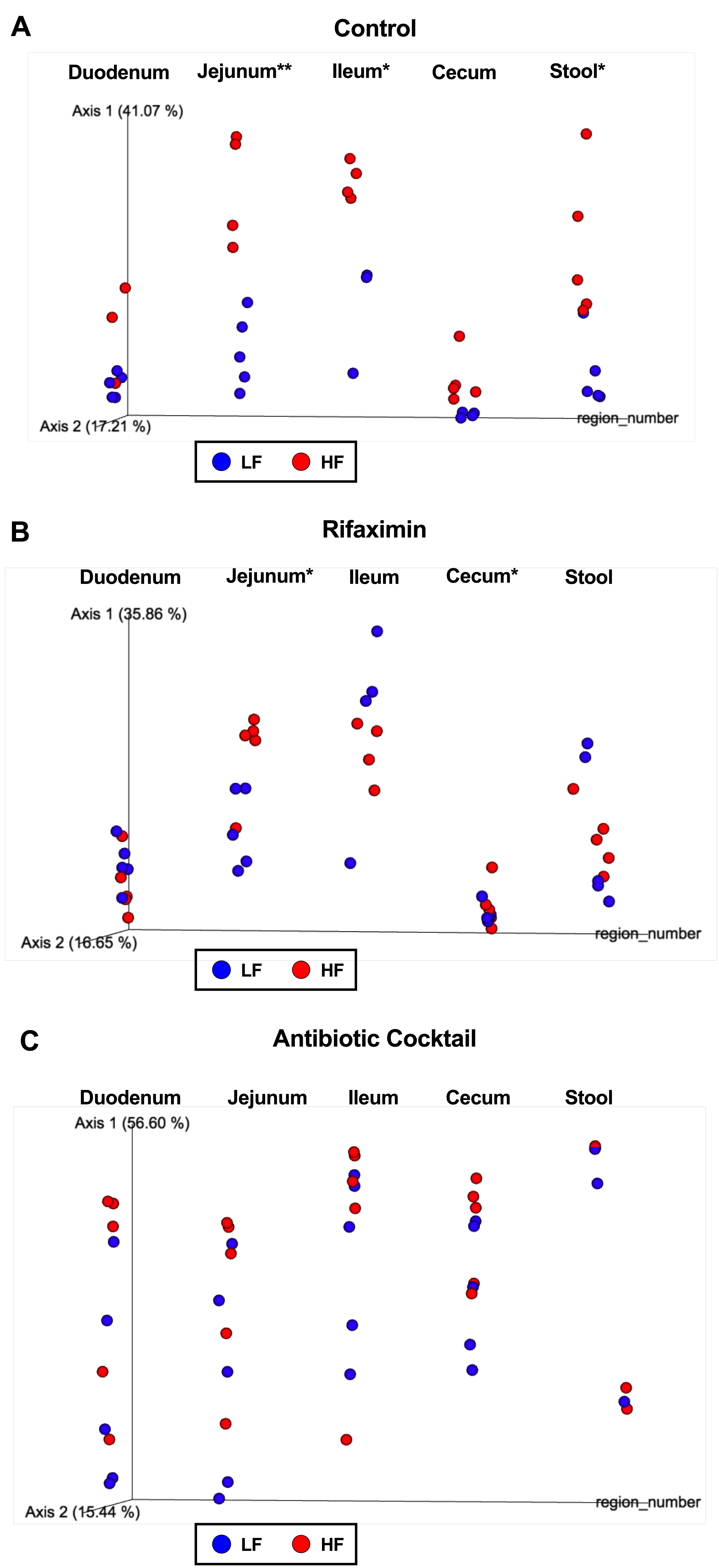


**Supplementary Figure 2.** **Antibiotics obscure the dietary impact on the gut microbiota along the length of the gut.** C57Bl6 mice were fed a low fat (LF) or high fat (HF) diet and treated with vehicle control, rifaximin or an antibiotic (Abx) cocktail for 4 weeks. A) A PCoA plot based on Bray Curtis dissimilarity index and on a forced axis for region is shown for all mice receiving control treatment. B) A PCoA plot based on Bray Curtis dissimilarity index and on a forced axis for region is shown for all mice receiving rifaximin treatment. C) A PCoA plot based on Bray Curtis dissimilarity index and on a forced axis for region is shown for all mice receiving antibiotic cocktail treatment. Adonis tests were performed to establish significant differences based on the effects of diet within each treatment condition, * p < 0.05, ** p < 0.01.

# Supplementary Tables

Supplemental Table 1. Two-way adonis results including diet and antibiotics as well as the interaction term between diet and antibiotics within each region of the gut.

|  | **Diet** | |  | **Antibiotics** | |  | **Diet-Antibiotics** | |
| --- | --- | --- | --- | --- | --- | --- | --- | --- |
| **Region** | R^2^ | P value |  | R^2^ | P value |  | R^2^ | P value |
| Duodenum | 0.0565 | 0.062 |  | 0.0737 | 0.001 |  | 0.0737 | 0.132 |
| Jejunum | 0.2228 | 0.001 |  | 0.0823 | 0.002 |  | 0.0823 | 0.092 |
| Ileum | 0.0562 | 0.139 |  | 0.1467 | 0.048 |  | 0.1467 | 0.061 |
| Cecum | 0.0445 | 0.03 |  | 0.0343 | 0.001 |  | 0.0343 | 0.218 |
| Stool | 0.0248 | 0.378 |  | 0.0762 | 0.002 |  | 0.0762 | 0.174 |

Supplemental Table 2. Pairwise adonis results for each GI region (R^2^ and adjusted p values are shown).

|  | **Duodenum** | | **Jejunum** | | **Ileum** | | **Cecum** | | **Stool** | |
| --- | --- | --- | --- | --- | --- | --- | --- | --- | --- | --- |
| **Diet Comparisons** | **R^2^** | **Adj. P value** | **R^2^** | **Adj. P value** | **R^2^** | **Adj. P value** | **R^2^** | **Adj. P value** | **R^2^** | **Adj. P value** |
| LF ctrl vs HF ctrl | 0.31 | 0.2438 | 0.63 | 0.0325 | 0.61 | 0.2063 | 0.23 | 0.0782 | 0.31 | 0.0625 |
| LF rfx vs HF rfx | 0.13 | 0.4455 | 0.30 | 0.0488 | 0.12 | 0.6058 | 0.25 | 0.0423 | 0.00 | 0.9810 |
| LF abx vs HF abx | 0.11 | 0.5190 | 0.17 | 0.1812 | 0.03 | 0.8290 | 0.23 | 0.1430 | 0.05 | 0.7018 |
|  |  |  |  |  |  |  |  |  |  |  |
| **Antibiotic Comparisons: LF** | **R^2^** | **Adj. P value** | **R^2^** | **P value** | **R^2^** | **Adj. P value** | **R^2^** | **Adj. P value** | **R^2^** | **Adj. P value** |
| Ctrl LF vs Rfx LF | 0.10 | 0.5190 | 0.25 | 0.0407 | 0.24 | 0.3800 | 0.57 | 0.0180 | 0.15 | 0.3082 |
| Ctrl LF vs Abx LF | 0.14 | 0.4455 | 0.39 | 0.0263 | 0.45 | 0.2063 | 0.68 | 0.0180 | 0.50 | 0.0625 |
| Rfx LF vs Abx LF | 0.10 | 0.5190 | 0.24 | 0.0788 | 0.12 | 0.4938 | 0.73 | 0.0180 | 0.42 | 0.0643 |
|  |  |  |  |  |  |  |  |  |  |  |
| **Antibiotic Comparisons: HF** | **R^2^** | **Adj. P value** | **R^2^** | **P value** | **R^2^** | **Adj. P value** | **R^2^** | **Adj. P value** | **R^2^** | **Adj. P value** |
| Ctrl HF vs Rfx HF | 0.37 | 0.2438 | 0.33 | 0.0717 | 0.43 | 0.2063 | 0.28 | 0.0438 | 0.26 | 0.0625 |
| Ctrl HF vs Abx HF | 0.26 | 0.3570 | 0.12 | 0.4840 | 0.09 | 0.6386 | 0.63 | 0.0180 | 0.06 | 0.7018 |
| Rfx HF vs Abx HF | 0.08 | 0.5190 | 0.21 | 0.0788 | 0.29 | 0.2750 | 0.85 | 0.0180 | 0.34 | 0.0625 |

| **Duodenum** | | | | | | | |
| --- | --- | --- | --- | --- | --- | --- | --- |
| **Family** | **FDR**  **p value** | **LF**  **Control** | **LF Rifaximin** | **LF Abx Cocktail** | **HF**  **Control** | **HF Rifaximin** | **HF Abx Cocktail** |
| unk_o_Bacteroidales_family | 0.0481 | 136894.8 | 163401.8 | 99554.8 | 115284.3 | 192109.6 | 53709.0 |
| Bacteroidaceae | 0.0481 | 76037.2 | 148867.0 | 87868.0 | 27359.3 | 173863.0 | 47727.0 |
| Streptococcaceae | 0.0481 | 85078.4 | 87014.8 | 289010.2 | 193537.7 | 47982.6 | 545261.2 |
| unk_Clostridiales_family | 0.0481 | 3920.2 | 6673.6 | 265.8 | 111.0 | 1403.4 | 562.4 |
| Ruminococcaceae | 0.0481 | 70075.4 | 64334.8 | 46918.6 | 33839.0 | 84306.2 | 21858.0 |
| Erysipelotrichaceae | 0.0481 | 175379.0 | 92705.6 | 79598.2 | 141085.3 | 83068.6 | 24870.0 |
| Verrucomicrobiaceae | 0.0481 | 142483.0 | 223419.4 | 187140.6 | 102089.7 | 220242.0 | 38277.8 |
| **Jejunum** | | | | | | | |
| **Family** | **FDR**  **p value** | **LF**  **Control** | **LF Rifaximin** | **LF Abx Cocktail** | **HF**  **Control** | **HF Rifaximin** | **HF Abx Cocktail** |
| Erysipelotrichaceae | 0.0455 | 322559.8 | 189688.8 | 48414.6 | 47047.25 | 115346.8 | 24394 |
| **Ileum** | | | | | | | |
| **Family** | **FDR**  **p value** | **LF**  **Control** | **LF Rifaximin** | **LF Abx Cocktail** | **HF**  **Control** | **HF Rifaximin** | **HF Abx Cocktail** |
| - | - | - | - | - | - | - | - |
| **Cecum** | | | | | | | |
| **Family** | **FDR**  **p value** | **LF**  **Control** | **LF Rifaximin** | **LF Abx Cocktail** | **HF**  **Control** | **HF Rifaximin** | **HF Abx Cocktail** |
| unk_o__Bacteroidales_family | 0.0077 | 159314.8 | 174460.4 | 76892.4 | 117763.2 | 168362.8 | 40260.2 |
| Bacteroidaceae | 0.0077 | 98672.3 | 193320.0 | 73110.8 | 95215.6 | 199497.2 | 44770.6 |
| Rikenellaceae | 0.0077 | 21101.3 | 7604.2 | 3343.2 | 20404.0 | 1494.4 | 1713.4 |
| Streptococcaceae | 0.0050 | 40695.5 | 34348.4 | 577311.4 | 90362.8 | 65653.6 | 739450.4 |
| unk_Clostridiales_family | 0.0227 | 5456.5 | 12669.8 | 2930.8 | 4690.6 | 135.6 | 3313.6 |
| Defluviitaleaceae | 0.0050 | 8992.5 | 0.0 | 119.0 | 4017.2 | 32.0 | 348.0 |
| Lachnospiraceae | 0.0077 | 176058.0 | 25506.8 | 17984.8 | 237737.4 | 79515.6 | 23126.0 |
| Peptostreptococcaceae | 0.0313 | 3138.8 | 58.2 | 651.6 | 26085.6 | 1753.2 | 3223.2 |
| Ruminococcaceae | 0.0113 | 75542.5 | 67562.4 | 21414.8 | 79302.0 | 97249.8 | 19367.0 |
| Alcaligenaceae | 0.0077 | 12014.3 | 36740.8 | 15685.4 | 20918.2 | 17184.6 | 4382.6 |
| Pseudomonadaceae | 0.0095 | 312.0 | 323.2 | 2392.6 | 271.4 | 139.6 | 3026.8 |
| Anaeroplasmataceae | 0.0313 | 503.8 | 3274.8 | 882.8 | 0.0 | 712.2 | 996.2 |
| Verrucomicrobiaceae | 0.0077 | 130687.3 | 347855.8 | 137811.2 | 213246.0 | 313023.4 | 70461.8 |
| **Stool** | | | | | | | |
| **Family** | **FDR**  **p value** | **LF**  **Control** | **LF Rifaximin** | **LF Abx Cocktail** | **HF**  **Control** | **HF Rifaximin** | **HF Abx Cocktail** |
| unk_o__Bacteroidales_family | 0.0363 | 75006.2 | 79862.8 | 9216.8 | 33778.2 | 114675.0 | 8524.6 |
| Anaeroplasmataceae | 0.0382 | 40.2 | 182.0 | 0.0 | 0.0 | 111.6 | 6.8 |

Supplemental Table 3. Kruskal-Wallis results by all treatment combinations within each region (taxa significantly different between diet and treatment groups based on FDR p < 0.05).
